# Genetic Diagnosis of Oculocutaneous Albinism Type 1A: A Novel *TYR* Variant

**DOI:** 10.1002/ccr3.70818

**Published:** 2025-08-25

**Authors:** Raghad N. Shihab, Rusul Thabit Hamid, Mostafa Neissi, Javad Mohammadi‐Asl, Motahareh Sheikh‐Hosseini, Elaheh Nekouei

**Affiliations:** ^1^ Cancer Researches Department, Iraqi Center for Cancer and Medical Genetic Research Mustansiriyah University Baghdad Iraq; ^2^ Department of Genetics, Khuzestan Science and Research Branch Islamic Azad University Ahvaz Iran; ^3^ Department of Genetics, Ahvaz Branch Islamic Azad University Ahvaz Iran; ^4^ Noor‐Gene Genetic Laboratory Ahvaz Iran; ^5^ Department of Medical Genetics, School of Medicine Ahvaz Jundishapur University of Medical Sciences Ahvaz Iran; ^6^ Pediatric Cell & Gene Therapy Research Center Tehran University of Medical Sciences Tehran Iran; ^7^ Department of Biomedical and Clinical Science Linköping University, University Hospital Linköping Sweden

**Keywords:** exome‐sequencing, oculocutaneous albinism, *TYR* gene, variant

## Abstract

Oculocutaneous albinism type IA (OCA1A) is a rare autosomal recessive disorder caused by variants in the TYR gene, resulting in complete loss of tyrosinase activity and absence of melanin production. In this study, we report a novel missense variant, *TYR* (NM_000372.5):c.143A>C (p.Gln48Pro), found within exon 1 and mapped to chr11: g.89178096A>C (GRCh38/hg38). This variant was identified through exome sequencing in a 4‐year‐old Iranian girl from a consanguineous family presenting with features of OCA1A. This discovery expands the mutational spectrum of OCA1 and underscores the importance of genetic screening in diagnosing rare inherited disorders. Future studies involving functional assays are necessary to elucidate the molecular mechanisms of this variant and its potential impact on melanogenesis.

**Summary**

- Early genetic diagnosis of OCA1A through exome sequencing enables accurate clinical management and timely genetic counseling, especially in consanguineous populations.
- The identification of a novel *TYR* (NM_000372.5):c.143A>C (p.Gln48Pro) variant highlights the value of genetic screening in guiding diagnosis, risk assessment, and reproductive planning.

## 1 Introduction

Oculocutaneous albinism (OCA) comprises a group of rare, genetically inherited disorders characterized by reduced or absent melanin pigmentation in the skin, hair, and eyes, often accompanied by visual abnormalities such as photophobia, nystagmus, and reduced visual acuity [1, 2, 3, 4]. Among the various subtypes, OCA type 1 (OCA1, OMIM #203100) is caused by variants in the *TYR* gene, which encodes tyrosinase, the enzyme responsible for the initial steps of melanin biosynthesis [5, 6]. OCA1 is further classified into OCA1A and OCA1B. OCA1A, the most severe form, results in complete loss of tyrosinase activity and a lifelong absence of melanin, whereas OCA1B permits partial enzyme function, allowing some pigmentation to develop over time [7, 8].

Differentiating OCA1 from other OCA types, particularly OCA2, is crucial for accurate genetic counseling. OCA2 (OMIM #203200), the most common form globally, is caused by variants in the OCA2 gene and typically presents with some pigmentation at birth that may increase over time. In contrast, OCA1A is characterized by complete absence of pigmentation from birth, more severe visual impairment, and a lack of pigment progression [5, 6]. Genetic testing is essential to distinguish between these types, especially when phenotypic overlap occurs.

In consanguineous populations, the prevalence of autosomal recessive disorders such as OCA is elevated, underscoring the need for precise molecular diagnosis for effective management and risk assessment [9, 10]. Exome‐sequencing has emerged as a powerful tool to uncover pathogenic variants in rare genetic conditions, offering a high diagnostic yield in familial cases [11, 12]. In this study, we used exome‐sequencing to identify the genetic cause of OCA1A in an Iranian child.

## 2 Methods and Materials

### 2.1 Patient Evaluation

A 4‐year‐old girl from a consanguineous Iranian family residing in Ahvaz, Iran, was evaluated for clinical manifestations of OCA1. A detailed medical history was obtained, and a comprehensive clinical examination was performed to assess dermatological, ophthalmological, and hair characteristics. Standardized photography was used to document physical features, including hair, skin, and eye color, along with ocular symptoms. The patient's response to light exposure and visual function were also examined. The ophthalmological examination included assessment of visual acuity, photophobia, and ocular deviations. A slit‐lamp examination was conducted to evaluate iris translucency, while fundoscopic examination was performed to identify potential retinal hypopigmentation. Additional dermatological assessments were carried out to document skin sensitivity and texture.

### 2.2 Sample Collection and Genomic DNA Extraction

Peripheral blood (5 mL) was collected from the proband and both parents in EDTA‐coated tubes. Genomic DNA was extracted from leukocytes using the QIAamp DNA Blood Mini Kit (Qiagen) per the manufacturer's instructions. DNA purity and concentration were assessed with a NanoDrop spectrophotometer (Thermo Fisher Scientific) and verified by agarose gel electrophoresis. Samples were stored at −20°C until analysis. Written informed consent was obtained from the parents.

### 2.3 Exome‐Sequencing

Exome‐sequencing was performed on the proband to identify pathogenic variants. Genomic DNA from peripheral blood was extracted and prepared using the SureSelect Human All Exon V7 kit (Agilent Technologies, USA). Sequencing on the Illumina NovaSeq 6000 platform generated 150 bp paired‐end reads with > 100× mean on‐target depth. Quality control was conducted with FastQC (v0.11.9), and high‐quality reads were aligned to GRCh38/hg38 using BWA‐MEM (v0.7.17). Duplicate reads were marked with Picard (v2.23.8), and variants were called following GATK (v4.2.6.1) best practices, including base recalibration and indel realignment. Variants were annotated with ANNOVAR (2020Jun07) and dbNSFP v4.2a, incorporating population frequency and predictive scores. Common variants (MAF > 1%) were excluded based on gnomAD v2.1.1 and 1000 Genomes data, unless reported as pathogenic in ClinVar or HGMD.

### 2.4 Variant Filtering and Prioritization Strategy

A stepwise filtering strategy was applied to prioritize pathogenic variants relevant to the proband's phenotype. Filtering retained exonic and canonical splice‐site variants with ≥ 20× read depth, Phred score ≥ 30, and functional impact (missense, nonsense, frameshift, or splice‐site altering). Synonymous variants without predicted splicing effects were excluded. Given the family's consanguinity, homozygous variants were prioritized according to an autosomal recessive model, with dominant‐acting albinism alleles considered if strongly implicated. Candidate variants were then curated using OMIM and HPO terms relevant to OCA.

### 2.5 Protein Structural Modeling

Protein structural modeling was performed using SWISS‐MODEL, a homology‐based structure prediction tool. Wild‐type and mutant *TYR* protein structures were generated to evaluate structural and conformational changes induced by the amino acid substitution. Model quality was assessed using standard structural validation parameters.

### 2.6 Polymerase Chain Reaction (PCR) and Sanger Sequencing

Candidate variant validation and segregation analysis were performed using Polymerase Chain Reaction (PCR) and Sanger sequencing. Primers flanking the target region were designed with Primer3 [12], and amplification was carried out using Phusion High‐Fidelity DNA Polymerase (Thermo Fisher Scientific) under standard cycling conditions. PCR products were purified with the QIAquick PCR Purification Kit (Qiagen) and sequenced using the BigDye Terminator v3.1 Cycle Sequencing Kit (Applied Biosystems). Sequencing products were purified with the BigDye XTerminator Kit (Applied Biosystems) and analyzed on an ABI 3730xl Genetic Analyzer (Applied Biosystems).

## 3 Results

### 3.1 Clinical Findings

**FIGURE 1:**
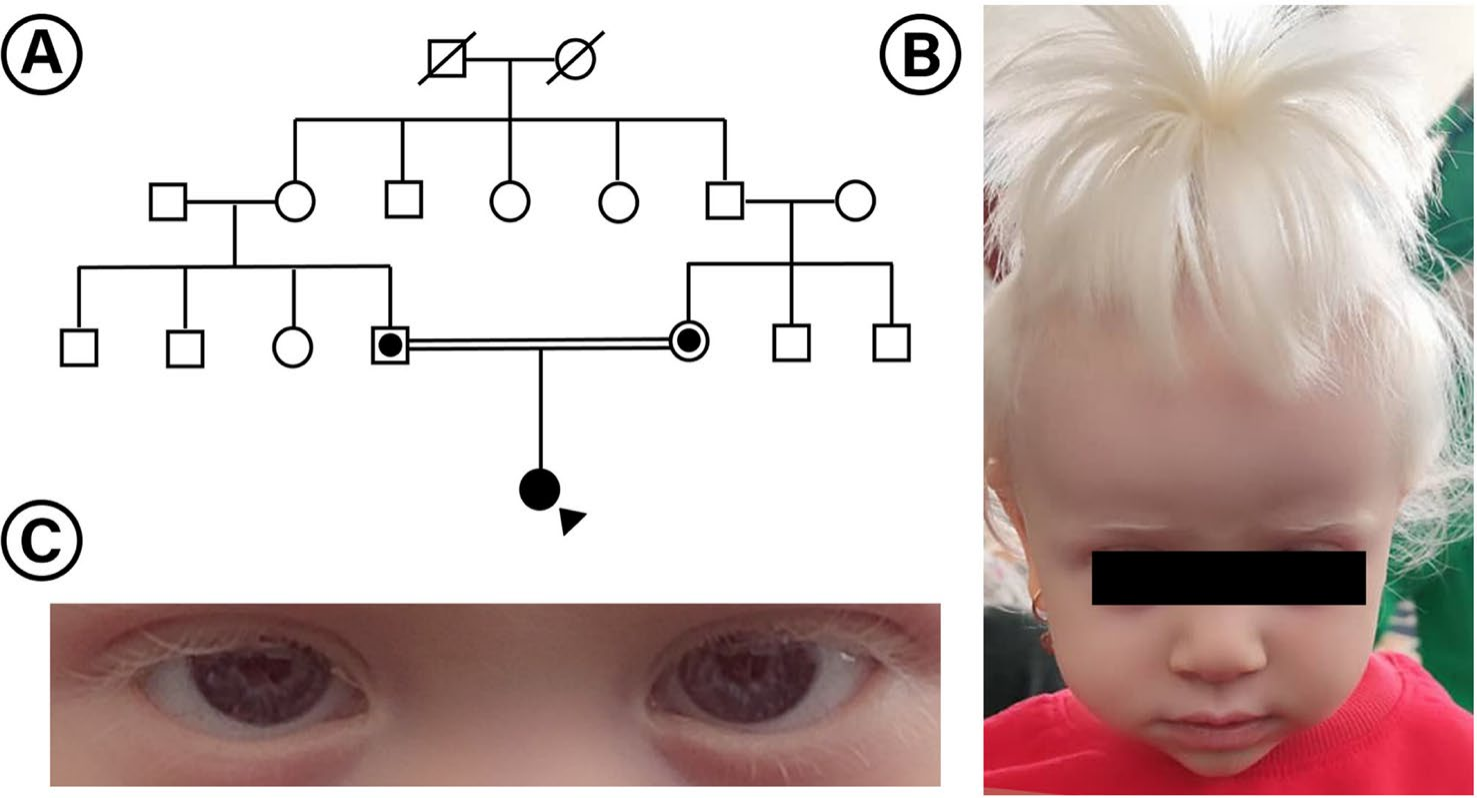
(A) Pedigree chart of the patient's family, indicating a consanguineous Iranian lineage. The diagonal slash denotes deceased individuals, circles represent females, squares denote males, and the filled symbol identifies the proband. The proband indicator (black triangle) points to the individual who underwent genetic analysis. Symbols with a central dot represent heterozygous carriers without clinical manifestation of the disease. The segregation pattern is consistent with autosomal recessive inheritance of OCA1. (B) Clinical image of the affected 4‐year‐old girl, displaying characteristic features of OCA1, including white hair and fair skin. (C) Close‐up of the patient's hypopigmented irises, revealing iris translucency and light‐colored eyes, consistent with OCA1‐associated ophthalmological findings.

The proband is a 4‐year‐old female born to a consanguineous Iranian couple (first cousins) (Figure 1A). She exhibited hallmark features of OCA1A, including complete absence of pigmentation in the skin, hair, and eyes from birth. Her hair was snow white, and her skin appeared uniformly pale with pronounced sun sensitivity (Figure 1B). No pigment development was observed over time, consistent with the lifelong absence of melanin production characteristic of OCA1A.

Ophthalmologic evaluation revealed significant photosensitivity, horizontal nystagmus, and mild esotropia. The irises were translucent with a blue‐gray hue and demonstrated a marked iris transillumination defect (Figure 1C); fundus examination revealed hypopigmentation of the retinal pigment epithelium. The patient's visual acuity was below age‐matched norms, and she exhibited photophobia even under indoor lighting. These findings are consistent with the severe ocular involvement characteristic of OCA1A.

A systemic evaluation was conducted to exclude syndromic forms of albinism. No abnormalities were identified in the cardiovascular, gastrointestinal, genitourinary, or neurological systems. The patient did not exhibit bleeding diathesis, immunodeficiency, or other features suggestive of Hermansky‐Pudlak syndrome or Chediak‐Higashi syndrome; thereby supporting a non‐syndromic diagnosis.

Both parents underwent detailed phenotypic assessment and showed normal pigmentation with no ocular abnormalities, aligning with their heterozygous carrier status. There was no reported history of similar features among other family members, though some relatives were not available for clinical assessment. No involvement of other organ systems was reported in the family history.

### 3.2 Identification of a Novel *TYR* Variant

**FIGURE 2:**
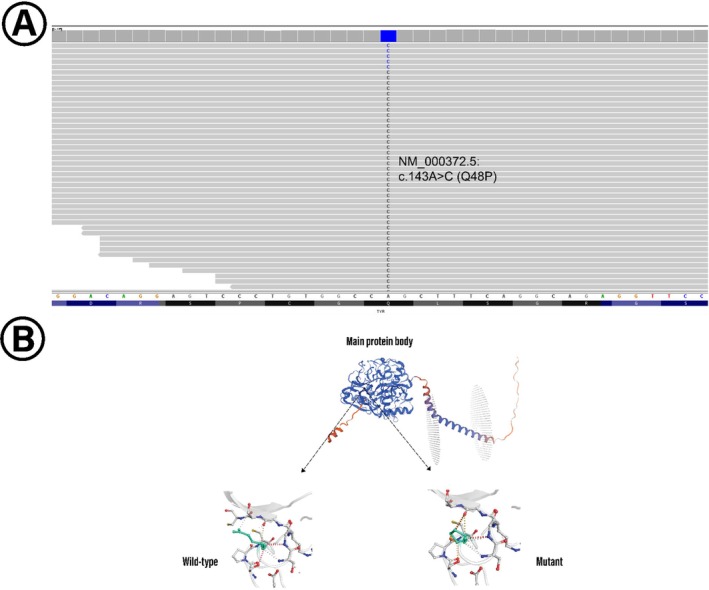
(A) IGV screenshot showing exome‐sequencing reads aligned to the *TYR* gene at the site of the NM_000372.5:C.143A>C (p.Gln48Pro) variant. All reads support the presence of the alternative allele [C], indicating homozygosity at the variant position in the proband. (B) Structural comparison of wild‐type and mutant TYR protein models, highlighting the impact of the p.Gln48Pro substitution. The overall tertiary structure is shown, with the affected region magnified in the lower panel. Wild‐type and mutant residues are depicted in light‐green stick representation alongside interacting residues. Hydrogen bonds and other interactions are marked by dashed lines, illustrating changes in the local structural environment.

Exome‐sequencing of the proband identified a novel missense variant, NM_000372.5:c.143A>C; p.Gln48Pro, in the *TYR* gene (GenBank: LC874533.1). This variant is located in exon 1 of 5 and corresponds to coding DNA position 143 of the 1590 bp coding sequence in the NM_000372.5 transcript, resulting in a glutamine‐to‐proline substitution at amino acid position 48. Genomic coordinates based on the GRCh38/hg38 reference genome indicate its location at chr11: g.89178096A>C, within chromosomal region 11q14.3. The variant was not detected in population databases and has not been previously reported in variant repositories. The variant was absent from population databases, including gnomAD and the 1000 Genomes Project, and has not been previously reported in any variant repositories. Specifically, the allele frequency in gnomAD was 0.0% across both founder and non‐founder populations. According to the PM2 criterion of the ACMG guidelines, a pathogenicity threshold of ≤ 0.15% is applicable for *TYR* gene variants. Known pathogenic *TYR* variants, such as chr11:89227904:C>A and chr11:89178093:G>A, are found at frequencies at or below this threshold. Therefore, the complete absence of the NM_000372.5:c.143A>C (p.Gln48Pro) variant in population databases strongly supports its rarity. No additional *TYR* variants were identified in the proband. According to OMIM and other gene‐disease association databases, *TYR* variants are linked to OCA1A, suggesting that this novel variant may be clinically relevant. To visually confirm the presence and zygosity of the variant, an Integrative Genomics Viewer (IGV) snapshot was generated at the variant site, illustrating consistent read support for the alternative allele (Figure 2A).

### 3.3 In Silico Pathogenicity Predictions

To evaluate the pathogenic potential of the *TYR* variant, we performed extensive computational analyses using multiple predictive algorithms integrated via dbNSFP. MutationTaster classified the variant as disease‐causing with a high confidence score (0.9209), and CADD (v1.6) yielded a PHRED‐like score of 24.1, indicating predicted deleteriousness. Additional algorithms, including BayesDel_addAF, FATHMM‐XF, MVP, MetaLR, and MetaSVM, provided varying levels of support for pathogenicity. While some predictors, such as AlphaMissense and MetaRNN, suggested a benign profile, others indicated moderate pathogenic potential. This divergence underscores the limitations of in silico prediction tools and highlights the need for functional assays to definitively determine the variant's biological impact.

Evolutionary conservation analysis further supports the clinical relevance of the variant. The affected glutamine at position 48 is highly conserved across vertebrates, as indicated by PhyloP100way, PhastCons100way, and SiPhy29way conservation scores. This suggests that the residue is functionally important, and its alteration may disrupt protein structure or enzymatic function. Homology‐based structural modeling corroborated this interpretation, revealing conformational changes introduced by the proline substitution, which likely compromise protein stability and local folding dynamics.

As shown in Table 1, among 13 predictive algorithms, 7 classified the variant as pathogenic or possibly pathogenic, while 6 indicated a benign or likely benign effect. Specifically, MetaLR, MetaSVM, MVP, and FATHMM predicted moderate pathogenicity, while CADD and MutationTaster further supported a deleterious impact. In contrast, AlphaMissense, MetaRNN, and EIGEN suggested a benign profile. This conflicting evidence points to a moderate pathogenic consensus and underscores the need for additional functional validation to clarify the variant's clinical significance.

**TABLE 1:** In silico pathogenicity prediction scores and effects.

Predictor	Classification	Score	Pathogenicity indicator
MutationTaster	Disease‐causing	0.9209	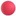
MetaLR	Pathogenic Moderate	0.9309	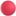
MetaSVM	Pathogenic Moderate	0.9617	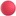
BayesDel addAF	Pathogenic Supporting	0.1911	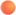
MetaRNN	Benign Supporting	0.3813	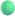
CADD	Possibly Pathogenic	16.45	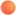 ^a^
MVP	Pathogenic Moderate	0.9929	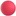
FATHMM	Pathogenic Moderate	−5.48	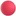
AlphaMissense	Benign Supporting	0.2825	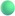
EIGEN	Benign Supporting	0.07699	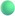
EIGEN PC	Benign Supporting	0.1432	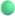
FATHMM‐XF	Benign Supporting	0.5135	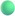
PhastCons100way	Highly Conserved	0.997	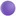
PhyloP100way	Highly Conserved	3.045	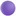
SiPhy29way	Evolutionary Constraint	10.5575	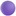
FitCons‐gm	Functional Constraint	0.5739	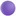

*Note:* 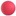 = Pathogenic; 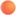 = Likely Pathogenic; 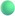 = Benign; 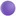 = Evolutionary/Functional indicators (non‐directly classifiable).

^a^ CADD scores above 15–20 are generally considered potentially pathogenic, depending on context.

The variant was interpreted according to the 2015 ACMG/AMP guidelines for sequence variant classification. Supporting criteria included PM2 (absent from population controls), PP1 (co‐segregation with disease in the family), and PP3 (multiple lines of computational evidence supporting a deleterious effect). PM5 was also applied, as other pathogenic missense variants have been reported at or near the same codon in *TYR*. However, due to the absence of functional studies or prior clinical reports specific to this variant, the final classification was designated as a Variant of Uncertain Significance (VUS).

The use of multiple in silico predictors was deliberate to enhance the robustness and reliability of pathogenicity assessment. Each algorithm evaluates distinct biological attributes—such as evolutionary conservation, protein structural stability, predicted functional disruption, and ensemble machine learning scores—providing complementary insights. Integrating outputs from diverse tools (e.g., MutationTaster, CADD, MetaSVM, BayesDel_addAF) reduces dependence on any single model and aligns with ACMG/AMP recommendations for computational evidence (PP3); thereby strengthening the rationale for the variant classification.

### 3.4 Protein Structure Analysis

The structural impact of the p.Gln48Pro variant in the TYR protein was assessed using homology modeling and molecular dynamics simulations. Comparative analysis of the modeled wild‐type and mutant protein structures revealed conformational differences, highlighting the potential effect of the amino acid substitution on protein function and intermolecular interactions.

A focused structural comparison at position 48 demonstrated significant alterations in local interactions (Figure 2B). The wild‐type glutamine, with its polar side chain, formed stabilizing hydrogen bonds and van der Waals interactions with neighboring residues. In contrast, the mutant proline, characterized by a rigid cyclic structure, disrupted these stabilizing interactions, resulting in a localized conformational shift. The loss of hydrogen bonding and steric constraints introduced by the proline residue was prominent, likely impairing protein stability and enzymatic function.

Additionally, analysis of the surrounding residues indicated alterations in the network of non‐covalent interactions, including the loss or weakening of hydrogen bonds and changes in hydrophobic packing. These structural deviations suggest a disruption in the protein's local folding and function, potentially affecting its overall enzymatic activity.

### 3.5 Validation and Segregation Analysis

**FIGURE 3:**
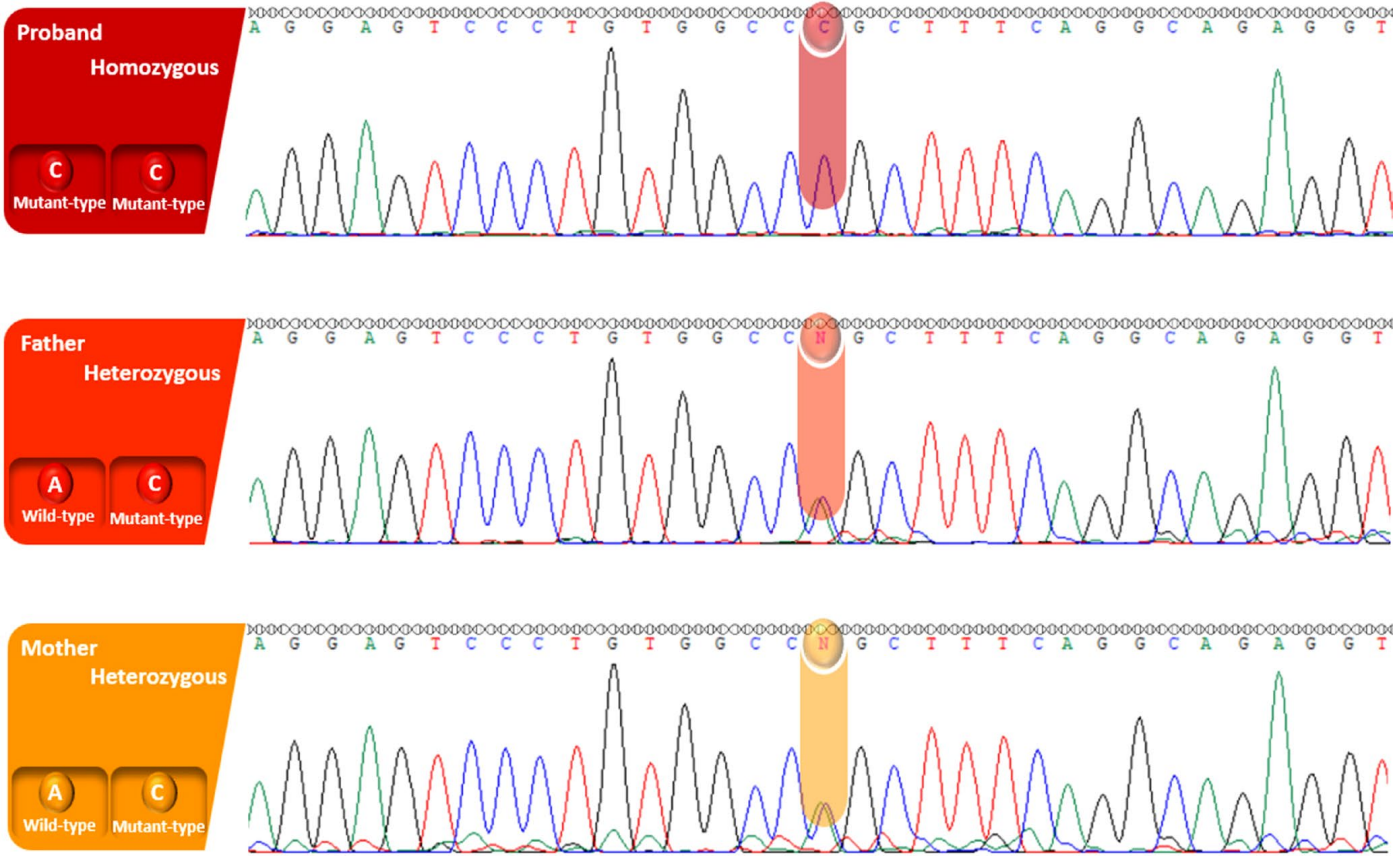
Sanger sequencing of the *TYR* c.143A>C (p.Gln48Pro) variant in the proband and parents. The proband is homozygous (A>C/C), while both parents are heterozygous (A/C), evidenced by overlapping peaks (denoted as “N” in chromatograms).

This analysis identified a novel homozygous missense variant, NM_000372.5:c.143A>C (p.Gln48Pro), located in exon 1 of the *TYR* gene. To confirm the presence and inheritance pattern of the variant, Sanger sequencing was performed on the proband and her biological parents. The proband was confirmed to be homozygous for the c.143A>C variant, while both parents were heterozygous carriers, consistent with their clinically unaffected status. This zygosity pattern supports an autosomal recessive mode of inheritance, which aligns with the consanguineous background of the family. Chromatograms confirming the segregation pattern are presented in Figure 3.

### 3.6 Follow‐Up

Given the autosomal recessive inheritance pattern of OCA1A, genetic counseling is essential to help families understand recurrence risks and reproductive options. In consanguineous populations, carrier screening of at‐risk relatives is particularly advisable. Prenatal genetic testing may be offered to guide early clinical decisions. Early ophthalmologic and dermatologic evaluations, along with sun protection strategies and low‐vision support, are critical to mitigate complications. Where available, preimplantation genetic testing (PGT) can assist in reducing recurrence risk in future pregnancies.

## 4 Discussion

The novel TYR missense variant c.143A>C (p.Gln48Pro) identified in our patient with OCA1A results in the substitution of glutamine with proline at position 48. This change is significant, as glutamine stabilizes protein conformation via hydrogen bonding, whereas proline—due to its rigid cyclic structure—disrupts local secondary structures [13, 14]. Given tyrosinase's central role in melanin biosynthesis, this structural alteration is likely to impair proper folding or enzymatic function, aligning with the patient's phenotype of complete pigment loss.

Several studies have examined how TYR gene variants affect melanin synthesis, tyrosinase function, and cellular processes underlying OCA1 pathogenesis. The TYR gene encodes tyrosinase, which catalyzes the conversion of tyrosine to L‐DOPA and then to DOPA‐quinone. Variants can cause misfolding, destabilization, or reduced activity, disrupting melanogenesis [7]. Spritz et al. identified multiple novel TYR variants in both OCA1A and OCA1B, with missense mutations often clustering near the amino terminus and in copper‐binding sites (CuA, CuB), directly impairing catalysis. For instance, p.Arg402Gln shows temperature‐sensitive activity, causing ER retention and reduced tyrosinase availability [15]. Wang et al. showed that some variants disrupt post‐translational modifications, such as glycosylation, essential for trafficking and stability; loss of glycosylation leads to mislocalization and premature degradation [16]. Mutations in conserved histidines within copper‐binding domains impair metal coordination, causing markedly reduced or absent melanin production [7, 15]. The novel missense variant NM_000372.5:c.143A>C (p.Gln48Pro) in exon 1 substitutes glutamine with rigid proline, likely distorting local structure and causing misfolding. Similar exon 1 variants, e.g., p.Cys89Ser, disrupt disulfide bonds and abolish activity [7]. Given its N‐terminal position and structural role, p.Gln48Pro likely impairs folding and function, leading to complete loss of melanin synthesis typical of OCA1A.

Table 2 summarizes previously reported TYR variants associated with OCA, compiled under strict inclusion criteria: (1) confirmed molecular diagnosis involving TYR variants, (2) detailed dermatologic and ophthalmologic phenotypic data, and (3) publication in peer‐reviewed literature. The table illustrates the genotype–phenotype spectrum in OCA1. Variants in exon 1 (e.g., p.Cys24Tyr, p.Gln48Pro, p.Cys89Ser) and exon 4 (e.g., p.Gly419Arg, p.Asp448Asn) are consistently linked to severe hypopigmentation and marked ocular involvement, suggesting disruption of core enzymatic domains or impaired protein folding. Conversely, variants such as p.Arg402Gln and p.Ser192Tyr, with partial enzymatic activity or temperature‐sensitive expression, are associated with milder, variable pigmentation. Compound heterozygotes often display intermediate phenotypes, modulated by the residual activity of the less deleterious allele, highlighting the complexity of TYR genotype–phenotype relationships. In our study, we identified a novel missense variant, NM_000372.5:c.143A>C (p.Gln48Pro), in exon 1, substituting glutamine with proline—a structurally disruptive change likely impairing protein folding and stability, consistent with the observed phenotype.

**TABLE 2:** Reported *TYR* gene variants (NM_000372.5) associated with OCA in previous studies.

Case	Sex	Age (years)	Ethnicity	Nucleotide change(s)	Amino acid change(s)	Coding impact	Location (exon)	Clinical symptoms	References
1.	♀	N/A^a^	Chinese	c.1A>G	p.(Met1Val)	Start loss	1	Typical OCA1A: severe hypopigmentation and foveal hypoplasia	Sun et al. [17]
2.	♂	9	Caucasian	c.25delC	p.(Leu9CysfsTer22)	Frameshift	1	Severe hypopigmentation and classic ocular signs	Spritz et al. [15]
3.	♂	0.5	Chinese	c.47C>A	p.(Ser16Tyr)	Missense	1	Initial depigmentation, some pigmentation development; visual deficits	Li et al. [18]
4.	♀	7	Chinese	c.71G>A/c.230G>A	p.(Cys24Tyr)/p.(Arg77Gln)	Compound heterozygous (Missense/Missense)	1/5	Complete depigmentation and severe ocular symptoms	Wang et al. [16]
5.	♀	19	Iranian	c.98A>C	p.(Lys33Thr)	Missense	1	OCA1 signs with reduced vision and foveal underdevelopment	Ghodsinejad Kalahroudi et al. [7]
6.	♀	9	Iranian	c.140G>A	p.(Gly47Asp)	Missense	1	Complete depigmentation and photophobia	Ghodsinejad Kalahroudi et al. [7]
7.	♀	Adult	Caucasian	c.229C>T/c.823G>T	p.(Arg77Trp)/p.(Val275Phe)	Compound heterozygous (Missense/Missense)	1/2	Light pigmentation and full ocular OCA1 profile	Spritz et al. [15]
8.	♂	Adult	Caucasian	c.232G>T/c.1336G>A	p.(Glu78Ter)/p.(Gly446Ser)	Compound heterozygous (Nonsense/Missense)	1/4	Complete depigmentation with severe vision loss	Spritz et al. [15]
9.	♀	Adult	Caucasian	c.238T>C/c.1336G>A	p.(Trp80Arg)/p.(Gly446Ser)	Compound heterozygous (Missense/Missense)	1/4	Severe OCA1A features with trace enzyme activity	Spritz et al. [15]
10.	♂	Adult	Caucasian	c.242C>T/c.649C>G	p.(Pro81Leu)/p.(Arg217Gly)	Compound heterozygous (Missense/Missense)	1/1	Complete albinism with profound visual impairment	Spritz et al. [15]
11.	♂	21	Iranian	c.265T>A	p.(Cys89Ser)	Missense	1	Classic OCA1A presentation with visual symptoms	Ghodsinejad Kalahroudi et al. [7]
12.	N/A	N/A	Iranian	c.286dupA	p.(Met96AsnfsTer73)	Frameshift	1	Full OCA1A phenotype with foveal hypoplasia	Khordadpoor Deilamani et al. [19]
13.	♂	24	Chinese	c.346C>T	p.(Arg116Ter)	Nonsense	1	Complete depigmentation with iris transillumination	Wang et al. [16]
14.	N/A	N/A	Russian	c.575C>A	p.(Ser192Tyr)	Missense	1	OCA, detailed phenotype not reported	Shchagina et al. [20]
15.	♂	39	Israeli Arab	c.757G>A	p.(Gly253Arg)	Missense	1	Severe hypopigmentation and reduced visual acuity	Spritz et al. [15]
16.	♂	15	Pakistani	c.826T>C	p.(Cys276Arg)	Missense	2	Albinism with strabismus and weak eyesight	Bibi et al. [21]
17.	♀	13	Pakistani	c.832C>T	p.(Arg278*)	Nonsense	2	Identical phenotype to sibling: milky skin, gray iris, nystagmus	Bibi et al. [21]
18.	♀	3.5	Caucasian	c.895C>A	p.(Arg299Ser)	Missense	2	Mild pigmentation with ocular signs	Spritz et al. [15]
19.	♀	18	Chinese	c.929dup	p.(Arg311LysfsTer7)	Frameshift	2	Reduced melanin, foveal and iris involvement	Wang et al. [16]
20.	♀	4	Chinese	c.985T>C	p.(Ser329Pro)	Missense	2	White features and nystagmus	Wang et al. [22]
21.	N/A	N/A	Iranian	c.996G>A	p.(Met332Ile)	Missense	2	Full OCA1A features with foveal hypoplasia	Khordadpoor Deilamani et al. [19]
22.	♂	1	Jamaican (Mixed Caucasian & Black)	c.1015A>G/c.1205G>A	p.(Ser339Gly)/p.(Arg402Gln)	Compound heterozygous (Missense/Missense)	2/4	Near‐total depigmentation and photophobia	Spritz et al. [15] and Shchagina et al. [20]
23.	♂	12	Iranian	c.1037G>A	p.(Gly346Glu)	Missense	3	Light hair/skin, optic nerve misrouting	Ghodsinejad Kalahroudi et al. [7]
24.	♂	Adult	Caucasian	c.1063G>C/c.1342G>A	p.(Ala355Pro)/p.(Asp448Asn)	Compound heterozygous (Missense/Missense)	3/4	Mild pigmentation and severe visual issues	Spritz et al. [15]
25.	N/A	N/A	Russian	c.1205G>A	p.(Arg402Gln)	Missense	4	OCA with no detail on phenotype	Shchagina et al. [20]
26.	♀	22	Iranian	c.1217C>T	p.(Pro406Leu)	Missense	4	Depigmented features with foveal hypoplasia	Ghodsinejad Kalahroudi et al. [7]
27.	N/A	N/A	Iranian	c.1255G>C	p.(Gly419Arg)	Missense	4	Complete OCA1A profile with iris signs	Khordadpoor Deilamani et al. [19]

^a^ Not applicable.

The role of missense variants in *TYR*‐related OCA is well documented, with specific amino acid alterations shown to interfere with tyrosinase activity. Structural disruptions caused by variants in exon 1 have been implicated in severe pigmentation deficiencies and visual impairments, as seen in reported cases [7, 15, 16, 17, 18]. Our analysis suggests that the p.Gln48Pro substitution may alter local protein conformation, likely encoding a defective enzyme that impacts function. Similar disruptions have been noted in other missense variants affecting key functional domains, further supporting the pathogenic nature of this alteration.

Computational modeling provided additional insight into the molecular consequences of this variant, revealing alterations in hydrogen bonding and local folding. The introduction of proline, a rigid, non‐polar residue, likely imposes steric hindrance, affecting protein integrity and resulting in impaired tyrosinase function. While enzymatic assays are necessary to confirm its precise functional impact, the observed clinical manifestations align with known *TYR* variants associated with a severe reduction in melanin synthesis.

Clinically, our patient showed features of profound melanin deficiency, including white hair, blue‐gray irises, and photosensitivity. Ophthalmological findings included nystagmus and photophobia, typical of defective tyrosinase function, along with mild strabismus—a less common but reported OCA1 feature [21]. Similar to previously described Iranian cases [7], the phenotype supports a link between exon 1 variants and disease severity. Our results highlight the critical role of exon 1 variants in TYR‐related OCA, with p.Gln48Pro representing a novel pathogenic change. Functional studies, including enzymatic assays and patient‐derived melanocyte models, are needed to clarify its effect on tyrosinase activity and melanin biosynthesis.

## 5 Conclusion

In this study, we identified a novel missense variant, NM_000372.5: c.143A>C (p.Gln48Pro), in the *TYR* gene from an Iranian patient with OCA1A. This finding expands the mutational spectrum of OCA and highlights the utility of exome sequencing in detecting rare genetic alterations, particularly in consanguineous populations. Our results underscore the importance of comprehensive genetic screening and counseling in the clinical management of OCA. However, future studies involving functional assays and larger cohorts are essential to confirm the biological impact of the p.Gln48Pro substitution, clarify its role in melanogenesis, and explore potential therapeutic strategies for *TYR*‐related albinism.

## Author Contributions

**Raghad N. Shihab:** investigation, writing – original draft. **Rusul Thabit Hamid:** investigation, writing – review and editing. **Mostafa Neissi:** conceptualization, investigation, writing – original draft. **Javad Mohammadi‐Asl:** investigation. **Motahareh Sheikh‐Hosseini:** investigation, writing – review and editing. **Elaheh Nekouei:** investigation.

## Consent

The family has provided written informed consent for this publication.

## Conflicts of Interest

The authors declare no conflicts of interest.

## Data Availability

The data that support the findings of this study are available from the corresponding author upon reasonable request.
